# Pomegranate Juice Augments Memory and fMRI Activity in Middle-Aged and Older Adults with Mild Memory Complaints

**DOI:** 10.1155/2013/946298

**Published:** 2013-07-22

**Authors:** Susan Y. Bookheimer, Brian A. Renner, Arne Ekstrom, Zhaoping Li, Susanne M. Henning, Jesse A. Brown, Mike Jones, Teena Moody, Gary W. Small

**Affiliations:** ^1^Center for Cognitive Neurosciences, Department of Psychiatry and Biobehavioral Sciences and Semel Institute for Neuroscience and Human Behavior, University of California, Los Angeles, 760 Westwood Plaza, Los Angeles, CA 90024, USA; ^2^Center for Human Nutrition, David Geffen School of Medicine, and the UCLA Longevity Center, University of California, Los Angeles, Los Angeles, CA, USA

## Abstract

Despite increasing emphasis on the potential of dietary antioxidants in preventing memory loss and on diet as a precursor of neurological health, rigorous studies investigating the cognitive effects of foods and their components are rare. Recent animal studies have reported memory and other cognitive benefits of polyphenols, found abundantly in pomegranate juice. We performed a preliminary, placebo-controlled randomized trial of pomegranate juice in older subjects with age-associated memory complaints using memory testing and functional brain activation (fMRI) as outcome measures. Thirty-two subjects (28 completers) were randomly assigned to drink 8 ounces of either pomegranate juice or a flavor-matched placebo drink for 4 weeks. Subjects received memory testing, fMRI scans during cognitive tasks, and blood draws for peripheral biomarkers before and after the intervention. Investigators and subjects were all blind to group membership. After 4 weeks, only the pomegranate group showed a significant improvement in the Buschke selective reminding test of verbal memory and a significant increase in plasma trolox-equivalent antioxidant capacity (TEAC) and urolithin A-glucuronide. Furthermore, compared to the placebo group, the pomegranate group had increased fMRI activity during verbal and visual memory tasks. While preliminary, these results suggest a role for pomegranate juice in augmenting memory function through task-related increases in functional brain activity.

## 1. Introduction

As people age, their risk for cognitive decline increases. An estimated 40% of people 65 years and older have age-associated memory impairment characterized by self-perception of memory loss and a standardized memory test score demonstrating lower objective memory performance compared with young adults [1–3]. While genetic factors play an important role in age-related memory decline, such nongenetic lifestyle factors as diet and exercise contribute as well [4]. In particular, a growing body of evidence indicates that accumulation of oxidative damage to macromolecules increases progressively during the aging process [5, 6] and contributes to neurodegeneration in Alzheimer's disease [7]. Epidemiological studies have suggested a link between antioxidant consumption and cognitive protection [8–11], and studies using a rodent model of Alzheimer's disease suggest that tau phosphorylation occurs as a compensatory response to oxidative stress [12]. Several clinical trials of antioxidant use in subjects with normal aging or Alzheimer's disease suggest memory benefits [13–15], while others yielded negative results [16–19]. 

While many fruits are rich in various antioxidants, including ascorbic acid, carotenoids, and phenolics, commonly consumed fruits show large differences in antioxidant capacity, as determined by the ferric reducing/antioxidant power (FRAP) assay [20]. In particular, there is special interest in the pomegranate polyphenols, which have been studied extensively in animal models of Alzheimer's disease [21, 22]. The emerging data on pomegranate fruits and their inherent polyphenols suggest positive benefits ranging from neuroprotective effects [23, 24] to staving off effects of senescent neurodegeneration in animal models of Alzheimer's disease [25–27]. 

Previously, we found that pomegranate juice has the highest antioxidant capacity among fruit juices using five different in vitro antioxidant assays [28]. In comparison, apple juice, which is low in antioxidant capacity, was less effective than pomegranate juice in affecting antioxidant capacity based on FRAP assays in 26 elderly subjects consuming pomegranate juice or apple juice over a 4-week period [29]. 

Overexpression of antioxidant enzymes or supplementation of some antioxidants appears effective in extending the life span in several animal models [5, 6], and a few studies have specifically found memory-enhancing effects of polyphenols in mice [30, 31]. However, evidence for memory enhancement from polyphenols in human-controlled trials is limited. One preliminary study [15] found beneficial effects of grape juice on memory in five older adults with age-associated memory impairment compared to seven placebo controls; to our knowledge this small study is the only placebo-controlled human trial of polyphenols that assessed memory change. Therefore, more well-controlled studies are needed to determine potential beneficial effects of antioxidants that occur naturally in various food sources and to elucidate the possible underlying mechanisms.

Moreover, studies that examine possible brain mechanisms of polyphenol treatment in humans are limited. One potential method for identifying mechanisms of recovery in drug trials is functional magnetic resonance imaging (fMRI). While structural MRI studies measure long-term changes in metrics, such as cortical thickness and volume [32–34], fMRI measures blood flow changes in response to memory challenge, which has proven to be effective in determining brain differences among subjects with Alzheimer's disease, mild cognitive impairment, or nondemented subjects at risk for Alzheimer's disease [35–40]. Further, fMRI has proven to be sensitive in measuring changes in neural activation patterns among older subjects in response to memory-enhancing drugs, such as donepezil [41] and memantine [42], with results showing increased activation or restoration of resting-state brain networks following treatment. One investigation used fMRI to examine the effects of antioxidants (flavonoids) in young healthy controls and demonstrated increased fMRI activity to cognitive challenge with flavonoid administration [43]; however, no studies to date have examined fMRI effects of antioxidant therapy in older adults with or without memory complaints. To address this knowledge gap, we explored potential neuroprotective effects derived from polyphenols in pomegranate juice in older volunteers with mild memory complaints in a placebo-controlled, randomized, and double-blind trial and measured three aspects: (1) metabolites of pomegranate juice using blood biomarkers; (2) effects of pomegranate juice on memory performance; and (3) evidence of functional MRI changes during memory activation. We hypothesized that pomegranate juice compared to placebo would increase metabolites, improve memory, and increase task-related brain activation in the left and right hemispheres for verbal and nonverbal memory tasks, respectively. Together these metrics provide preliminary evidence of bioavailability, clinical efficacy, and brain mechanisms of pomegranate juice in older adults with memory complaints.

## 2. Methods

### 2.1. Subjects

Thirty-two volunteers were initially enrolled into the study out of 39 who were screened; of these, 28 completed the clinical trial (Table 1). Subjects were recruited via advertisements through local newspapers, flyers, posters, lectures, and word-of-mouth. We specifically recruited nondemented, older right-handed subjects with self-reported age-related memory complaints. The subjects did not carry a diagnosis of mild cognitive impairment (MCI) and were screened using the mini-mental state examination (MMSE). Subjects randomized to placebo versus control groups did not differ in mean age, percentage of women, mean MMSE scores, or baseline memory performance (Table 1). Potential subjects were excluded who had documented neurological, psychiatric, or major medical conditions, including heart disease, diabetes, and gastrointestinal disease, that might affect cognitive function or interfere with study procedures. We also excluded subjects who routinely ate more than three servings per day of fruits and vegetables or were taking vitamin supplements, any medication, or dietary supplement that interferes with the absorption of polyphenols. Potential subjects participating in regular vigorous exercises other than ordinary daily walks or who were unwilling to maintain a sedentary lifestyle during the 28-day study were excluded, as were those for whom MRI was contraindicated (e.g., claustrophobia, dental braces, or implanted ferromagnetic or electronic devices). Seven potential subjects were screened out based on one or more of these exclusion criteria.

Subjects were instructed that they would be removed from the study if they failed to follow the prescribed low polyphenol diet during the one-week run-in period based on self-report in-person interview with a study dietitian. The study was approved by the UCLA Institutional Review Board; all subjects gave informed consent to participate in all procedures and were reconsented for followup evaluations. Funding for this study was provided by the UCLA Center for Human Nutrition from a “various donors” account. The investigators were blind to the contributors to this fund, and there was no communication or input from donors or commercial entities at any stage of the study.

### 2.2. General Procedures

Following phone screening, volunteers participated in an initial clinical evaluation, which included an explanation of the study procedures, consent, medical screening, MMSE, and routine CBC and chemistry panel. Prior to the intervention phase, each subject met with a registered dietitian and was instructed on a low polyphenol diet, which required that they restrict their intake of several fruits and vegetables, onions, tea, chocolate, and dried beans, for one week prior to the baseline visit and for the duration for the study. At baseline, subjects underwent neuroimaging and neurocognitive testing (described later). We also acquired blood samples to perform the antioxidant assay and to verify compliance with the protocol. Subjects were then randomized to either the pomegranate or placebo groups; investigators and subjects were blind to group membership. 

After these procedures, subjects were given a one-week supply of 8-ounce containers of pomegranate or placebo juice with instructions to consume 8 ounces of juice daily. The pomegranate drink was the commercial Pom Wonderful product. The flavor-matched drink contained sugar, citric acid, and food color to match the calorie, taste, and color of the juice. Each week, subjects returned their empty juice containers to ensure compliance, picked up their juice supply for the following week, and met briefly with the dietitian to ensure compliance with the low polyphenol diet. At the end of the study (day 28), subjects again underwent neuroimaging, cognitive assessment, and blood draws. 

 A placebo drink using sugars and natural colorings and flavoring to imitate the taste and appearance of pomegranate juice was used. The research personnel were blinded as to the assignment of subjects to placebo or pomegranate juice. 

### 2.3. Behavioral Testing Procedures

Each subject underwent fMRI and brief memory testing immediately prior to beginning the trial and following 28 days of pomegranate juice or placebo. Subjects received a memory testing battery and underwent functional MRI scanning on the same day. Memory tests administered were the Buschke-Fuld selective reminding task [44], from which we calculated two performance measures, total items recalled and consistent long-term retrieval; an experimental unrelated word pair associates learning task [36], based on the Wechsler memory scale paired associates learning [45] in which we used identical procedures to that used in the fMRI scan but with different stimuli. We also obtained behavioral data from the MRI tasks administered in the scanner (described below). Of the 28 subjects completing the study, 26 had behavioral data on both occasions (two did not participate in one testing session; their remaining behavioral data were eliminated from analysis). Subjects were administered one of two alternate forms of each task, with the order of forms counterbalanced across subjects, to avoid practice effects. To assess changes in verbal memory, we compared performance on total number of items recalled (TR) on the Buschke-Fuld selective reminding task (sum of all items recalled on all trials) and on the consistent long term retrieval (CLTR) score (sum of items recalled in consecutive trials without item repetition). The TR score emphasizes immediate recall, while CLTR depends upon memory consolidation processes. To determine memory change, we first examine the *t*2 versus *t*1 difference in scores within groups using a paired *t*-test. To determine whether the treatment effects differed between groups, we then tested for differences between the pomegranate juice and placebo groups using a 2-group unpaired *t*-test. Because memory performance in this age range is highly variable, we used time 2 minus time 1 difference scores as our dependent variables. At baseline, there were no differences between groups on either TR performance (two-tailed *t* = .8; *P* ≥ .4) or CLTR (two-tailed *t* = .5; *P* > .9). For this reason our analysis did not covary baseline performance; instead we performed a *t*-test of group differences on the change scores. 

### 2.4. Plasma Biomarkers

Plasma obtained from all subjects prior to and at the end of the trial was tested for TEAC and urolithin A-glucuronide. The trolox-equivalent antioxidative capacity (TEAC) was determined using 2′, 2′-azinobis(3-thylbenzo thiazoline-6-sulfonic-acid) diammonium (ABTS) radical cations oxidized with by adding solid manganese dioxide in 75 mmol/L sodium/potassium phosphate (Na/K) buffer of pH 7. Trolox (6-hydroxy-2,5,7,8-tetramethylchroman-2-carboxylic acid), a water-soluble analog of vitamin E, was used as an antioxidant standard. Plasma was diluted 1 : 20 in Na/K buffer pH 7. Diluted plasma samples were assayed in four replicates. TEAC values were calculated from the trolox standard curve (0–350 *μ*mol/L) and expressed as trolox equivalents (in mmol/L) [46]. Plasma urolithin A-glucuronide was measured using high performance liquid chromatography mass spectrometry (LC-MS) as described previously [47]. Of all biomarker data across the two time points, only three data points were missing (one placebo subject, TEAC Day 0; two PJ subjects, UA-Glu day 28); the calculations were therefore based on the remaining subjects. Plasma TEAC was equivalent between groups at baseline (*P* > .17; see Table 1), so we calculated the percent change in TEAC for each subject and compared the change scores.

### 2.5. MRI Procedures

Functional and structural images were collected on a 3T Siemens Trio MRI scanner using a 32-channel head coil. Structural MRI was collected using a *T*1-weighted magnetization prepared rapid gradient echo (MPRAGE) volumetric scan sequence using axial acquisition slicing, TR = 2.3 sec, TE = 2.93 msec, field of view = 256 × 256 mm^2^, flip angle = 8°, resolution 160 × 192 × 192, voxel size = 1.5 mm^3^. Scans were reviewed to rule out subjects with brain abnormalities (evidence of stroke, structural lesion, or atrophy indicative of dementia). 

Functional MRI was collected using a *T*2*-weighted echoplanar pulse sequence with a TR = 2.5 sec, TE = 35 msec, field of view = 200 × 200 mm, flip angle = 90°, collecting whole brain images with resolution = 64 × 64 × 28, voxel size = 3 mm^3^. For localization, image registration, and spatial normalization, we collected a coplanar, matched-bandwidth, spin-echo echoplanar scan on each subject (TR = 5000 ms, TE = 33 ms, 128 × 128 matrix size). Experimental tasks (described below) were presented via magnet compatible video and audio system (Resonance Technologies, Inc, Northridge, CA), which included video goggles with lenses for vision correction optimized for each subject. Responses were recorded and made using a button press. Stimuli were presented via a Macintosh computer running MATLAB.

For the functional MRI tasks, subjects received computer training of tasks prior to scan sessions. Two memory tasks reported previously by our group were presented during scanning: a verbal memory task (unrelated word-pair associates learning) [36] and a visual memory task (spatial navigation) [48]. Following 28 days of intervention (*t*2), the fMRI and behavioral testing procedures were repeated and compared to *t*1 using alternate forms.

### 2.6. Verbal Memory Task

The unrelated word-pair task was identical to that previously reported by Bookheimer et al. [36]. Briefly, subjects were presented seven pairs of unrelated words (e.g., table flower) in six learning blocks, interspersed with baseline fixation and retrieval blocks. During each learning block, subjects heard each word pair presented over two seconds with a two-second interval before the next pair (total block length = 28 sec). Following a 20-second fixation baseline, they performed the retrieval task in which they were presented the first word in each pair and asked to generate the matching word. The word lists were matched for word frequency, imageability, and form. Behavioral performance was collected at the end of each scan and in an alternate form administered outside the scanner to obtain a learning curve. The total scan length was 9 minutes. 

### 2.7. Visual Memory Task

 The spatial navigation task involved learning and recalling routes on a virtual map, while subjects play the role of a “taxi driver.” During the encoding phase, subjects watched and rewatched two sets of video clips. In each video, subjects first saw the name of the imaginary town, such as “Johnsberg,” then for 15 second blocks they saw a first-person view of “driving” to a virtual town laid out in 5 × 5 block grids, to a storefront destination with unique characteristics for specific storefronts, stopping for 3 seconds before proceeding to a new store within the same town starting from a different location. After each city, subjects performed a 30-second distractor task where they pressed a left or right button in response to a probe. This was repeated while subjects learned 6 cities in total. 

The fMRI data were then collected during memory retrieval in three conditions. Subjects were asked to remember one of three characteristics about each storefront for the separate tasks. Two conditions focused on the physical features of the environment (specific stores-item task and physical placement-location task). In condition 1, participants were asked to identify an appearance change of the store (*identification)*, such as signs, colors, and doors. In condition 2, they were asked to identify the spatial location change of the store within the town, relative to other buildings (*location identification*), respectively. Condition 3 required subjects to identify the store (*source*) for a specific item that might be purchase there, requiring the participant to encode the association between an item and a spatial location. For this source task, subjects had to recall whether the store was in the first or second town (memory for object-location associations). Only the latter condition tapped into the associative memory processes. The order of the three conditions was counterbalanced across subjects. 

### 2.8. fMRI Analysis

All functional data were analyzed using FSL version 4.1.4 (FMRIB's Software Library, http://www.fmrib.ox.ac.uk/fsl). Preprocessing included motion correction to the mean image, spatial smoothing (Gaussian kernel FWHM = 5 mm), and high-pass temporal filtering (*t* > 0.01 Hz). Subjects with head motion exceeding an average of 2 voxels were removed from analysis. Twenty-three (13 pomegranate juice, 10 placebo) subjects were able to complete the visual memory fMRI task without significant head motion, while for the verbal memory task, eight pomegranate juice and nine placebo subjects were able to complete both scanning sessions with high quality images. Each functional scan was registered to its corresponding coplanar high-resolution image with rigid body transformations and to the MNI152 standard brain using nonlinear transformation (12 degrees of freedom). Additionally, a high-pass temporal filtering of 100 sec was applied to the functional images. Individual preprocessing and statistical analysis of each subject's functional scan was run using FSL FMRI Expert Analysis Tool (FEAT); FEAT was then used to analyze data at the group level. Without strong a priori hypotheses about region-specific effects of polyphenols (other than presuming left versus right hemispheric changes for verbal and visual memory tasks, resp.), we corrected for multiple comparisons across the entire brain using FSL's FEAT, correcting at the cluster level. FEAT uses an FWE (family-wise error) cluster-based thresholding using random field theory to correct for multiple comparisons across space. Thresholding defines contiguous clusters; each cluster's estimated significance level from Gaussian random field-theory is compared with the cluster probability threshold. 

First, for both the verbal and visual memory tasks, we contrasted task versus baseline for each subject at each time point (before and after treatment). To minimize the number of comparisons, we restricted our analysis to learn versus baseline and recall versus baseline for the verbal memory task and task versus baseline for each of the three visual memory conditions, (item, source, and spatial). Next, for each task, the individual subject data were entered into a second-level analysis of within-group means for each contrast. To test the hypothesis that pomegranate juice and placebo groups differed in *t*2 versus *t*1 activation patterns, between-group, between time point contrasts were generated using an ANOVA model in FSL, cluster-corrected at *Z* > 2.0, *P* = .05, and corrected for multiple comparisons. Finally, for each task, the individual subject data were entered into additional analyses of within-group between time point, and between-group within time point. 

## 3. Results

### 3.1. Peripheral Biomarkers

**Figure 1:**
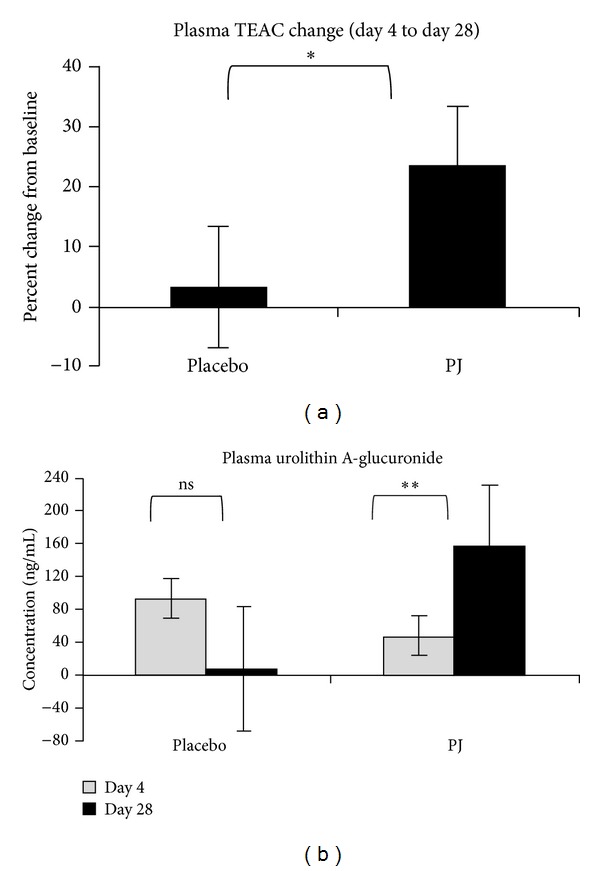
Metabolite results. (a) The pomegranate juice group had significantly higher change in TEAC after day 28 compared with baseline than the placebo group (*unpaired *t* = 2.8, df = 25,  *p* < .05). (b) Plasma urolithin A-glucuronide significantly increased in the pomegranate juice group (**paired *t* = 3.93, df = 13, *p* < .00064), but not in the placebo group (paired *t* = 1.7, df = 11, *p* > .09).

At baseline, the groups did not differ in antioxidant levels (TEAC; *t* = 1.37, *P* > .17). The pomegranate juice group had significantly higher TEAC values after day 28 compared with baseline than the placebo group (unpaired *t* = 2.8, df = 25,  *P* < .05; Figure 1). Similarly, plasma urolithin A-glucuronide increased significantly in the pomegranate juice, but not in the placebo group (unpaired *t* = 3.75; df = 24, *P* < .001). To evaluate compliance, we identified the number of participants whose urolithin A-glucuronide values increased versus remained the same or less after day 28 across the groups. Two of 13 placebo subjects had increased urolithin A-glucuronide values after 28 days versus 11 of 14 pomegranate juice subjects, a difference that was significant (chi-square = 4.8; *P* < .03).

### 3.2. Memory Performance

The within-group analysis of changes following treatment found that, on average, the pomegranate juice group performed significantly better at *t*2 compared with *t*1 (using alternate test forms; two-tailed *t* = 2.8, *P* = .017). For the placebo group, there was no significant difference between *t*2 and *t*1 performance scores (two-tailed *t* = 1.1, *P* > .3). 

**Figure 2:**
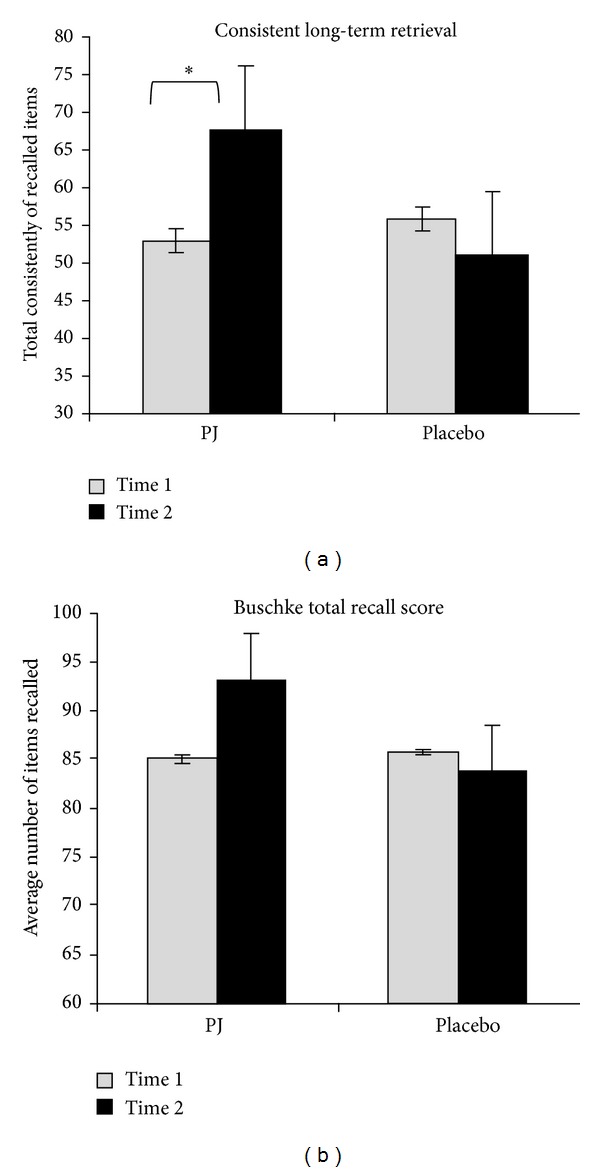
Buschke selective reminding test results. (a) On the total recall measure, the *t*2 versus *t*1 change in memory scores was significantly greater in the pomegranate juice group compared to the placebo group (mean change, number of items recalled: PJ = 7.7, placebo = −2.77; *t* = 2.3; *P* = .029). (b) Similarly, on the consistent long-term retrieval score the pomegranate juice group recalled more items compared to the placebo group (mean change, number of items consistently recalled: PJ = 15.2, placebo = −9.7; *t* = 2.4; *P* = .022).

Between groups, the *t*2 versus *t*1 improvement in memory scores was significantly greater in the pomegranate juice group compared to the placebo group on the total recall measure (two-tailed *t* = 2.3; *P* = .029; Figure 2). Similarly on consistent long-term retrieval the pomegranate juice group recalled more items compared to the placebo group (two-tailed *t* = 2.4; *P* = .022; Figure 2).

### 3.3. Imaging Results

Performance on the visual and verbal memory tasks during scanning did not differ between groups at either timepoint (all *P* values > .1); thus subsequent analyses of activation differences could not be attributed to differential performance. 

#### 3.3.1. Visual Memory fMRI Task

 Looking at within-group means for both groups and at both time points, we found significant activation in visual pathways including bilateral occipital cortex extending into temporal fusiform and parahippocampal gyrus, as well as activation in subcortical region across the three task conditions, consistent with visual memory processing and spatial navigation [48]. 

**Figure 3:**
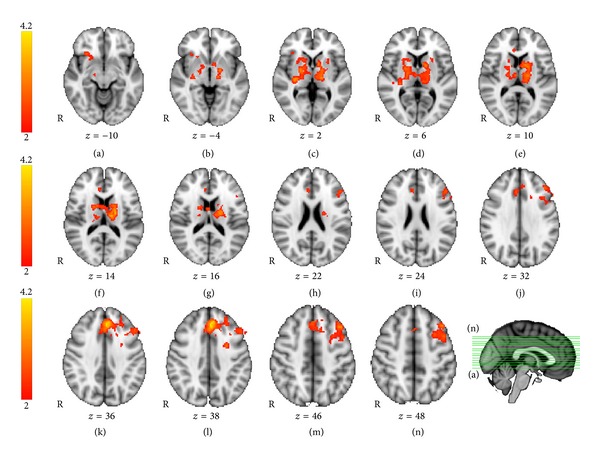
Visual memory fMRI task: between-groups *T*2 versus *T*1 ANOVA. Regions showing greater activation for *t*2 > *t*1, for the pomegranate juice group > placebo group (group by time interaction), were found bilaterally in the basal ganglia and thalamus, including caudate, putamen, and pallidum (ANOVA, *Z* > 2.0, *P* = .05, corrected for multiple comparisons). Additional regions significant at an uncorrected threshold (*Z* > 1.7) are listed in Table 2.

To test the hypothesis that brain activity during a visual memory task increased for the treatment group exclusively, we first performed a whole brain, voxel-wise ANOVA comparing the PJ and control groups on the *t*2 versus *t*1 contrast, corrected for multiple comparisons and thresholded at *Z* > 2.0. Results of the ANOVA are shown in Figure 3; coordinates of active brain regions are listed in Table 2. The pomegranate group showed greater fMRI activation than the placebo group in the *t*2 versus *t*1 contrast, located bilaterally in regions of the basal ganglia and thalamus. At a lower threshold of *Z* = 1.7 (*P* < .05), additional regions in the left inferior frontal gyrus and left middle frontal gyrus were recruited. In contrast and as predicted, no brain regions were more active in *t*2 versus *t*1 for the placebo group compared to the pomegranate group (no voxels exceeded threshold).

**Figure 4:**
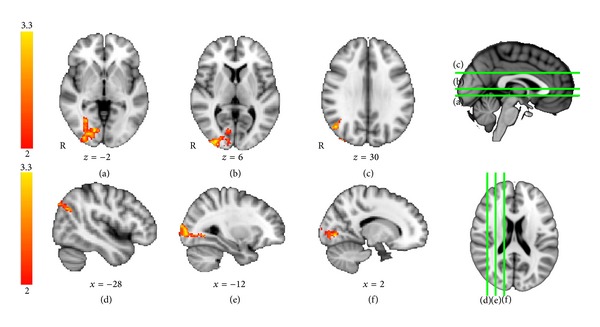
Visual memory fMRI task: between-groups *T*2. Activations for time point, *t*2, for pomegranate > placebo. For the visual memory task, at *t*2, between groups, pomegranate > placebo, there were significant activations in right lateral occipital cortex and fusiform gyrus (unpaired *t*-test, *Z* > 2.0, *P* = .05, corrected for multiple comparisons).

To further examine the effects of the PJ intervention on visual memory, we performed a secondary analysis comparing the PJ versus placebo groups for the visual memory versus control contrast at *t*2 only. This analysis directly compares visual memory processing between groups after treatment. The results of this analysis are shown in Figure 4. Using unpaired *t*-tests for comparing patterns of activation during the posttreatment scan between groups, the pomegranate group showed significantly greater activation than the placebo group in right occipital and right fusiform regions, extending into parahippocampal cortex (Figure 4). These regions are typically engaged during visual navigation memory tasks, indicating greater recruitment of visual memory regions in the treatment group compared with the placebo subjects. In contrast, and as expected, the placebo group did not activate any brain regions significantly more than the pomegranate group (no voxels exceeding threshold).

#### 3.3.2. Verbal fMRI Memory Task

**Figure 5:**
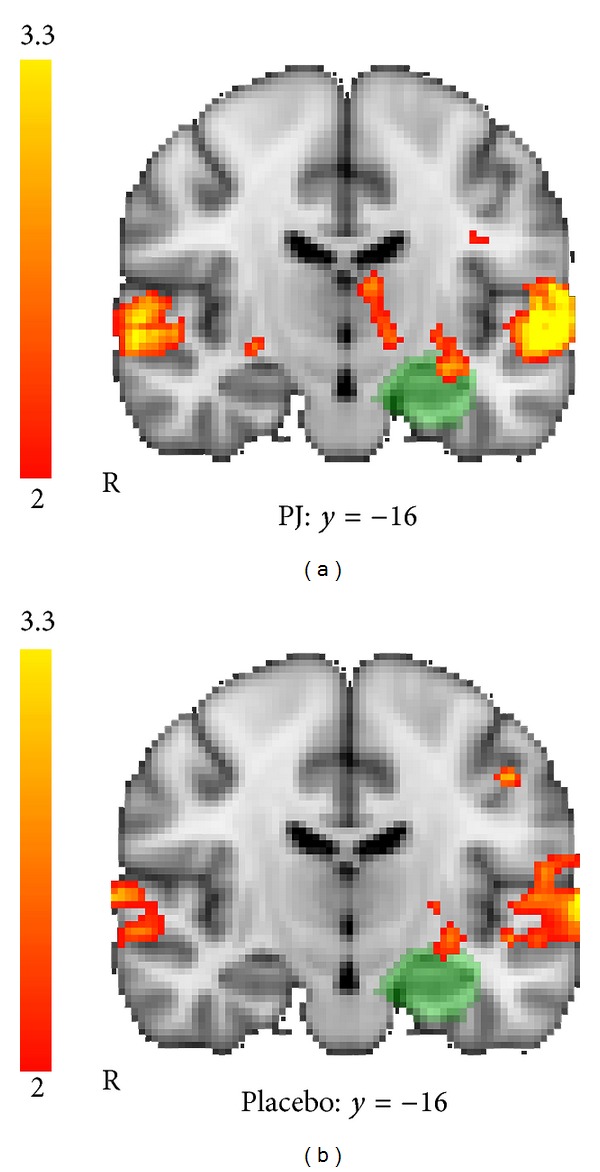
Verbal memory fMRI task: within-group activations at *t*2. Comparing the within-group results for time point *t*2, there were activation similarities and differences in the placebo group ((a): top) and the pomegranate group ((b): bottom). Only the pomegranate group recruited the hippocampus, bilaterally (group mean: placebo, *Z* > 2.0, *P* = .05, corrected for multiple comparisons, pomegranate, *Z* > 2.0, *P* = .05, corrected for multiple comparisons).

 We first identified brain regions activated during the verbal memory task compared to the baseline condition across all subjects and time intervals. Consistent with prior studies, the task verbal memory activated a wide network of brain regions, including bilateral occipital cortex, frontal lobe, and medial temporal lobe and subcortical regions including thalamus, putamen, and the hippocampus. Of note, for the verbal task, the pomegranate group activated basal ganglia, thalamus, and hippocampus in the posttreatment session and the placebo group did not (Figure 5). 

**Figure 6:**
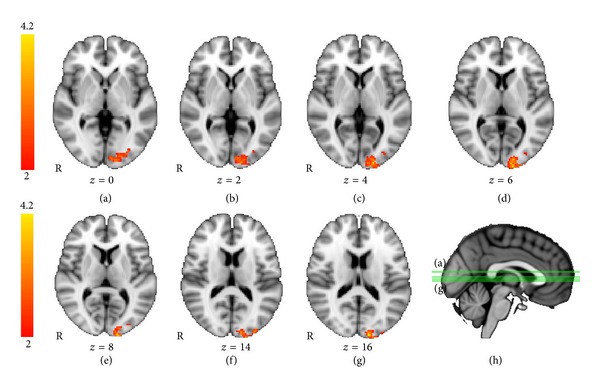
Verbal memory fMRI task: between-groups *T*2 versus *T*1 ANOVA. Regions showing greater activation for *t*2 > *t*1, for pomegranate group > placebo group, were found in the left hemisphere in occipital polar regions (ANOVA, *Z* > 2.0, *P* = .05, corrected for multiple comparisons).

We then compared the group by time interaction of fMRI activations for the PJ versus control group differences using ANOVA, identifying brain regions that showed greater fMRI activation signal after treatment, for *t*2 versus *t*1. For the verbal learning versus control contrast, we found that the PJ group showed significantly greater increases in activation compared to the placebo group for *t*2 versus *t*1. Regions showing differences were found in the left hemisphere, including regions of the left occipital lobe and left fusiform gyrus (*Z* > 2.0, *P* = .05, corrected for multiple comparisons), shown in Figure 6 and listed in Table 3. By contrast, but as expected, there were no regions in which the placebo group showed greater activation than the pomegranate juice group for *t*2 > *t*1. 

**Figure 7:**
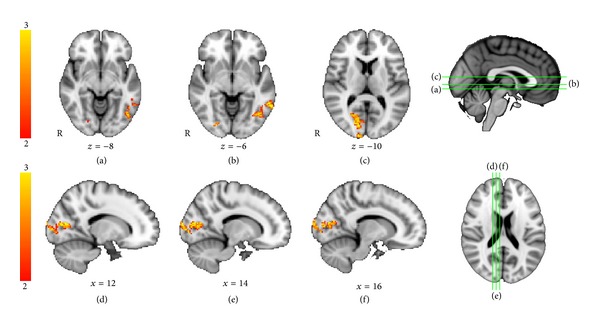
Verbal memory fMRI task: activations for the pomegranate group for *t*2 > *t*1. For the pomegranate group in the verbal memory task, there were significant activations between sessions, in the left inferior temporal gyrus and right occipital lobe (paired *t*-test between time points, *Z* > 2.0, *P* = .05, corrected for multiple comparisons). No regions were more active for *t*2 versus *t*1 in the placebo group.

To further examine treatment-related effects, we then examined regions of brain activity greater for *t*2 versus *t*1 within groups using paired *t*-tests. Only the pomegranate group showed significant differences in activation in the posttreatment session compared to pretreatment for any brain regions for the learn versus rest condition. Before treatment, the PJ group showed significantly greater fMRI activation in the left inferior temporal gyrus and right occipital lobe compared to the first session (Figure 7). As predicted, the placebo group did not show any regions with significantly greater activation in the second session compared to the first. It is important to note that between groups differences in change over time could be due either to increases in the PJ group or decreases in the placebo group at *t*2 relative to time 1. Within the PJ group, there was greater activation in *t*2 compared to *t*1 and no regions showed the reverse effect; thus the PJ group clearly demonstrated an increase in MRI activation following treatment. However, by examining the reverse contrast (time 1 > time 2), we found that the placebo group only also showed a decrease in fMRI activation at *t*2. Therefore, the ANOVA results appear to reflect both increases in the PJ group and decreases in the placebo group following intervention. 

## 4. Discussion

**Figure 8:**
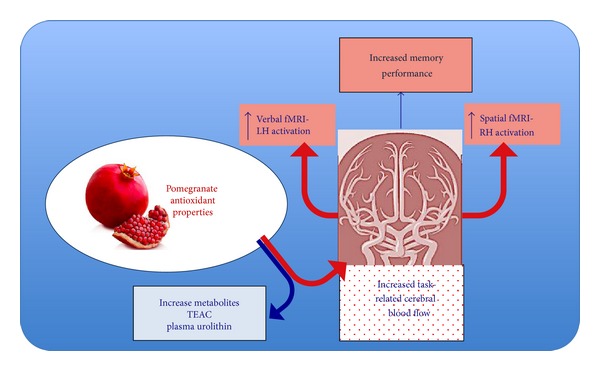
Summary of study results. Our data suggest that the antioxidant properties of pomegranate produce increased task related cerebral blood flow, with lateralized effects for verbal versus visual memory challenge, which in turn increased cerebral blood flow facilitates memory performance. Metabolic measures confirm the increase in polyphenols among the experimental group.

Our results support the hypothesis that polyphenols derived from pomegranate juice may improve memory in older persons with age-related memory decline.  Figure 8 summarizes our major findings integrating the hypothesized mechanism for effects of pomegranate juice on polyphenol metabolites, task-related cerebral blood flow, and memory performance. Peripheral measures of TEAC and urolithin A-glucuronide indicated that our subjects were compliant with their regimen and that the dose administered was sufficient to increase polyphenol metabolites. Moreover, memory assessments indicated that subjects who drank 8 ounces of pomegranate juice for a month experienced significant improvements on a sensitive measure of memory performance. As in fMRI studies of pharmacological interventions in memory-impaired subjects, the pomegranate juice intervention activated only memory-related brain regions, with no increases in the control group and no decreases in activation following intervention in the pomegranate juice group. Taken together, these findings support the hypothesis that drinking pomegranate juice may increase task-related brain activation and improve memory ability in older adults with preexisting mild memory complaints.

These findings are also consistent with animal studies showing positive effects of polyphenols on memory laboratory animals; previous studies have shown that combating oxidative stress through berry extract supplementation attenuates age-related cognitive deficits in aged rodents [26, 27, 49, 50]. Joseph and coworkers found that such supplementation reversed age-related deficits in working memory performance in the Morris water maze [49]. Other studies have shown that blueberry supplementation was effective in reversing cognitive declines in object recognition [51] Possible mechanisms for this effect of polyphenol supplementation include antioxidant, direct effects on signaling to enhance neuronal communication [50], and the ability to buffer against excess calcium [50]. These animal studies showing putative polyphenol antioxidant effects on cognitive led to our choice of pomegranate juice as an intervention, given the high polyphenol antioxidant properties of pomegranates [52]. 

The changes in fMRI activity in the pomegranate group are consistent with results seen on pharmacological trials in patients with mild cognitive impairment and Alzheimer's dementia. It is striking that both the verbal and nonverbal memory tasks showed virtually exclusive increases in activity following intervention (there were some parietal increases for placebo). Saykin and colleagues [41] found that short-term treatment with the cholinesterase inhibitor donepezil enhanced frontal circuitry activity in patients with amnestic mild cognitive impairment, and this increase in activation was related to improved cognition. Such increases may occur because the subjects can better focus on the cognitive task (increased cognitive effort) or alternatively may indicate greater task-related increases in cerebral blood flow. This fMRI method cannot distinguish between these possibilities; however, changes in task strategy tend to produce both task-related increases and decreases in fMRI signal [53, 54], whereas an hypothesis of increased blood flow responses would tend to predict only fMRI increases. Our data show evidence that the PJ group showed increases in fMRI activation at *t*2 that were greater than those for placebo, with no regions greater for the placebo group at time 2. However we also found some regions in which the placebo group showed reduced activation at *t*2 compared to *t*1. Thus the group by time interaction appears to reflect both increases in the experimental and decreases in the control group. Reduction in activation with repeated testing in fMRI is a common finding (e.g., [55]); however we can only conclude that there is *relatively* greater fMRI activation among the PJ group as we do not have absolute measures of cerebral blood flow.

Although the duration of our study was short, there is evidence in the animal literature suggesting that antioxidant use can affect cerebral blood flow, including task-related CBF, acutely and over short time intervals as in the present study. For example, Huang et al. [56] measured CBF, BOLD response to forepaw stimulation, and oxygen metabolic rate (CMRO2) in rats treated with the antioxidant methylene blue USP; they found increases in task-related BOLD response and CBF under normal breathing conditions; Bitner et al. [57] found that antioxidant carbon clusters restored CBF in an experimental traumatic brain injury model in rate under hypotension. The antioxidant black cumin oil administered over 10 weeks improved spatial cognition in a water maze task in rats, a task reminiscent of our spatial navigation task [58]. One recent study in humans found an increase in BOLD activation during a simple attention-switching task in subjects after 5 days of treatment with flavanol-rich cocoa [43]. In addition, they performed a second, preliminary study (*N* = 4) of acute flavanol administration during arterial spin labeling (ASL) MRI, which provides a quantitative measure of cerebral blood flow, finding increased CBF following flavanol administration. Together, these studies suggest that antioxidants administered either acutely or chronically increase cerebral blood flow and in one animal study also improved spatial memory [55]. 

It is also notable that the regional distribution of increased activations was largely specific to each task (i.e., left hemisphere for verbal memory, right hemisphere for visual memory). Of the three conditions in the visual spatial navigation task, the intervention significantly affected bold activation pattern in the source memory task. The source task placed particular emphasis on episodic memory, requiring subjects to learn and retrieve novel associations between specific items and the context in which they were encoded (i.e., episodic memory). Similarly, the verbal memory task was designed to tap associative encoding in the verbal domain. In the verbal task, there were increases in left hemisphere regions after treatment, and the pomegranate group showed activation in the temporal lobe in the posttreatment session that was not apparent in the placebo group. Associative, episodic memory tasks rely particularly on temporal lobe systems, which are critically involved in both age-related memory decline and Alzheimer's disease. 

The two measures of the Buschke selective reminding task are thought to assay different memory processes. In this test, subjects hear a long list of words and repeat back as many as they can recall; on subsequent trials they are reminded only of those words they did not recall on the last trial (hence, “selective” reminding). The total score reflects all recalls across up to 10 trials until all words are recalled consistently. By contrast, the consistent long-term retrieval score only counts items once they are consistently recalled for all subsequent trials (without reminders) and thus appears to depend upon consolidation processes mediated by the hippocampus. While the scores are not entirely independent (high scores on total recall require consistent retrieval), they are also not fully redundant, as it is possible to have recall scores of up to 80 (approximately the mean of our subjects' performance) while never retrieving a memory item consistently. The subjects in this study improved on both measures suggesting that the intervention may affect cognition broadly. Because our cognitive battery was limited in this preliminary study, however, we cannot tell if other aspects of cognition are affected by pomegranate juice. Thus, future studies should examine the effects of pomegranate juice or extract using a more comprehensive cognitive battery of tests.

Based on this work and the work on animals, we hypothesize that polyphenols (and other antioxidants) enhance resting and task-related cerebral blood flow. Increased metabolic activity should improve cognitive processing efficiency, in this case during memory tasks, increasing memory performance. If correct, we would predict a correlation between the magnitude of increased task-related activation in these brain regions and the degree of memory improvement after treatment. In our study, the number of subjects completing the fMRI procedures without head motion was smaller than that of those completing the memory tests; with the reduced sample size we were unable to make these direct correlations. Further studies with larger samples will allow us to test this model more directly. 

Other limitations of this study include the small sample size and brief treatment duration. Thus our results should be considered preliminary and should be validated in a larger scale, multicenter study. Because the functional MRI measure is a relative one and not a direct measure of cerebral blood flow, reduced base-line blood flow could provide an alternative explanation for the results. Differences between groups in the *T*2 versus *T*1 comparison could be due to either increases in task-related blood flow relative to *T*1 in the pomegranate group or to decreases in blood flow at *T*2 in the placebo group. Future studies could measure cerebral blood flow quantitatively using either H_2_^15^O positron emission tomography or arterial spin-labeled MRI. In this study our regional hypotheses were limited to hemispheric specificity for verbal versus visual tasks, given the lack of prior studies in humans. Accordingly we corrected our results for multiple comparisons across the whole brain, which may have been overly conservative. Future studies with larger N's could target specific regions of interest in relation to treatment-related memory changes. 

In summary, this preliminary randomized placebo-controlled, double-blind trial of pomegranate juice in older adults with mild memory complaints suggests that 8 ounces of pomegranate juice taken daily over one month improve a sensitive measure of verbal memory and alter neural activity during a visual source memory task. The presence of polyphenol metabolites validated compliance with the experimental regimen and efficacy of pomegranate juice in releasing polyphenols. fMRI results suggest that the mechanisms of change may involve increases in task-related cerebral blood flow, particularly during associative encoding tasks. Future studies are needed to validate these results within larger samples and determine the long-term effects of pomegranate juice on a range of comprehensive cognitive functions.

## Figures and Tables

**Table 1:** Demographic and clinical characteristics of subjects at baseline.

Characteristic	Mean (SD)	
	Pomegranate (*n* = 15)	Placebo (*n* = 13)
Mini-mental state examination	28.0 (1.5)	27.8 (1.5)
Age, y	63.1 (8.0)	62.0 (7.8)
Female, no. (%)	11 (73.3)	10 (76.9)
TEAC baseline	1712 (299)	1927 (461)
Buschke selective reminding test		
Recall	85.0 (11.7)	86.5 (12.5)
Consistent long-term retrieval	52.8 (19.9)	55.8 (25.5)

**Table 2:** Spatial memory fMRI activation clusters. ANOVA results showing the coordinates of maximally activated voxels of clusters for pomegranate juice group > placebo group for *t*2 greater than *t*1 (Z > 2.0, corrected P = 0.05). No regions were found for placebo > pomegranate for *t*2 > *t*1.

Spatial memory task	MNI coordinates (mm)			
	*x*	*y*	*z*	*Z* stat
*Z* > 2.0				
Subcortical regions				
Right thalamus	18	−14	8	2.60
Right pallidum	22	−4	2	2.98
Right putamen	30	−20	0	3.51
Left thalamus	−12	−14	10	3.44
Left pallidum	−18	−4	−2	3.34
Left caudate	−12	4	10	3.06

*Z* > 1.7				
Frontal Lobe				
Left paracingulate gyrus, superior frontal gyrus	−4	36	38	4.23
Left middle frontal gyrus	−38	24	44	3.19
Left middle frontal gyrus	−26	2	40	3.1
Left precentral gyrus	−44	0	52	3.06
Left inferior frontal gyrus, pars opercularis	−42	12	28	2.78
Left middle frontal gyrus, frontal pole	−46	32	28	2.75
Subcortical				
Right putamen	30	−20	0	3.49
Right pallidum	26	−20	0	3.25
Left pallidum	−18	−6	0	3.15
Left Thalamus	−16	−14	12	3.67
Left Thalamus	−14	−18	0	3.39

**Table 3:** Verbal memory fMRI activation CLusters. ANOVA results showing the coordinates of maximally activated voxels of clusters for pomegranate juice group > placebo group for *t*2 greater than *t*1 (*Z* > 2.0, corrected *P* = 0.05). No regions were found for placebo > pomegranate for *t*2 > *t*1.

Verbal memory task	MNI coordinates (mm)			
	*x*	*y*	*z*	*Z* stat
Brain region				
Occipital lobe				
Left occipital pole	−14	−98	16	3.97
Left fusiform gyrus	−18	−90	−4	3.55
Left lingual gyrus	−16	−76	−6	2.82
Left lateral occipital	−32	−80	−2	2.54
